# A Biomimetic Multifunctional Nanoframework for Symptom Relief and Restorative Treatment of Acute Liver Failure

**DOI:** 10.1021/acsnano.4c00173

**Published:** 2024-01-31

**Authors:** Ruibing Feng, Yu Fan, Xinya Zhang, Lanmei Chen, Zhang-Feng Zhong, Yitao Wang, Hua Yu, Qing-Wen Zhang, Guodong Li

**Affiliations:** † State Key Laboratory of Quality Research in Chinese Medicine, Institute of Chinese Medical Sciences, University of Macau, Macao SAR 999078, P. R. China; ‡ Macao Centre for Research and Development in Chinese Medicine, State Key Laboratory of Quality Research in Chinese Medicine, Institute of Chinese Medical Sciences, University of Macau, Macao SAR 999078, P. R. China; § Zhuhai UM Science and Technology Research Institute, Zhuhai 519031, P.R. China; ¶ The Marine Biomedical Research Institute of Guangdong Zhanjiang, School of Ocean and Tropical Medicine, Guangdong Medical University, Zhanjiang, Guangdong 524023, P.R. China

**Keywords:** acute liver failure, mesenchymal stem cell, biomimetic nanoparticle, N-acetylcysteine, rhein

## Abstract

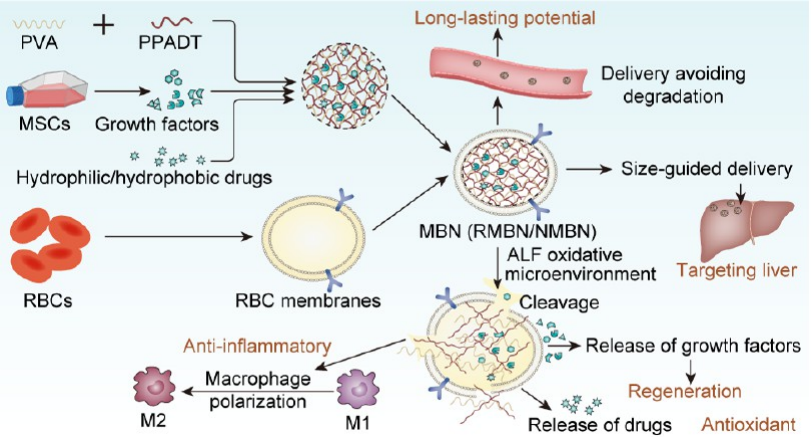

Acute liver failure (ALF) is a rare and serious condition characterized by major hepatocyte death and liver dysfunction. Owing to the limited therapeutic options, this disease generally has a poor prognosis and a high mortality rate. When ALF cannot be reversed by medications, liver transplantation is often needed. However, transplant rejection and the shortage of donor organs still remain major challenges. Most recently, stem cell therapy has emerged as a promising alternative for the treatment of liver diseases. However, the limited cell delivery routes and poor stability of live cell products have greatly hindered the feasibility and therapeutic efficacy of stem cell therapy. Inspired by the functions of mesenchymal stem cells (MSCs) primarily through the secretion of several factors, we developed an MSC-inspired biomimetic multifunctional nanoframework (MBN) that encapsulates the growth-promoting factors secreted by MSCs via combination with hydrophilic or hydrophobic drugs. The red blood cell (RBC) membrane was coated with the MBN to enhance its immunological tolerance and prolong its circulation time in blood. Importantly, the MBN can respond to the oxidative microenvironment, where it accumulates and degrades to release the payload. In this work, two biomimetic nanoparticles, namely, rhein-encapsulated MBN (RMBN) and N-acetylcysteine (NAC)-encapsulated MBN (NMBN), were designed and synthesized. In lipopolysaccharide (LPS)/d-galactosamine (D-GalN)-induced and acetaminophen (APAP)-induced ALF mouse models, RMBN and NMBN could effectively target liver lesions, relieve the acute symptoms of ALF, and promote liver cell regeneration by virtue of their strong antioxidative, anti-inflammatory, and regenerative activities. This study demonstrated the feasibility of the use of an MSC-inspired biomimetic nanoframework for treating ALF.

Acute liver failure (ALF) is a rare but severe condition characterized by massive hepatocyte death and liver dysfunction in patients who do not have a history of liver disease.^1^ The disease is most often caused by drugs, chemical poisons, or bacterial or viral infections.^2^ At present, clinical diagnosis and treatment approaches available for ALF are scarce.^3^ While liver transplantation is the gold standard for treating ALF, donor availability and transplant rejection remain major challenges.^4^ Therefore, alternative therapeutic or regenerative strategies for treating ALF are urgently needed.

In recent years, various types of nanoparticle-based delivery systems for controlled and sustained drug release, the transportation of insoluble drugs, and targeted therapy have been reported for the treatment of human diseases, including liver diseases.^5,6^ However, their controlled release properties and targeting efficiency are unsatisfactory in complicated biological environments, partly due to their inherent immunogenicity, poor stability and dispersion, and rapid clearance *in vivo*.^7−9^ Therefore, enhancing the specificity and prolonging the circulation time of drugs and reducing their aggregation at nontarget sites are highly important for the clinical translation of nanopreparations. In recent years, the development of cell membrane-coated nanoparticles for high-efficiency and low-toxicity treatment has achieved great success.^10,11^ Notably, it has been reported that nanoparticles coated with erythrocyte membranes, which contain membrane protein complexes that are crucial for immune tolerance, can prolong the circulation time of nanomaterials in blood and thus improve their therapeutic effects.^12^ Moreover, erythrocyte membrane-coated biomimetic nanomaterials with a size of ca. 200 nm are also retained in organs such as the liver, which is important for ALF treatment.^13^

Moreover, ALF is a disease with a high acute fatality rate. Therefore, timely management of acute symptoms is crucial to avoiding the systemic and irreversible damage it causes. The use of biomimetic nanomaterials for the targeted delivery and controlled release of antioxidative and anti-inflammatory drugs has been an effective strategy for relieving ALF symptoms.^14^ In addition to alanine transaminase (ALT) and inflammatory cytokines, reactive oxygen species (ROS) are considered ideal early biomarkers for ALF.^15,16^ The accumulation of ROS can induce necrosis and hepatocyte damage, causing damaged hepatocytes to release ALT into the blood.^17^ Moreover, ROS can trigger hepatic macrophages to generate proinflammatory cytokines, such as interleukin 1β (IL-1β), interleukin 6 (IL-6), and tumor necrosis factor-α (TNF-α).^17^

The biomimetic multifunctional nanoframework is a highly engineered structure designed to mimic biological processes at the nanoscale. These frameworks are typically created to perform multiple functions, such as targeted drug delivery, environmental remediation, and the simulation of natural tissue for medical applications. For instance, Vijayan et al. reported a biomimetic nanoframework for the sustained delivery of recombinant decorin from nanofiber dressings to potentially obstruct scar formation during the process of wound healing.^18^ Li et al. developed a biomimetic multifunctional lignocellulosic nanoframework for sustainable environmental remediation.^19^ In recent years, therapies based on mesenchymal stem cells (MSCs) and their derived microvesicles have emerged as promising strategies for attenuating ALF in various animal models. Moreover, a large number of MSC-based clinical trials, either ongoing or completed trials, can be found in the database of the U.S. National Institutes of Health.^13,20^ However, the disadvantages of cell therapy, including the requirement for stringent storage conditions, low efficacy due to lung filtration, lack of therapeutic effects, safety issues related to the immune response, and the requirement for repeated intravenous administration, have restricted its application.^21−23^ Notably, recent studies have demonstrated that MSCs function primarily through the secretion of several factors.^24^ For example, Cheng et al. presented an MSC/red blood cell (RBC)-inspired nanoparticle using the biocompatible polymer polylactic-*co*-glycolic acid (PLGA) for the treatment of carbon tetrachloride-induced ALF in mice. The results showed that MSC growth-promoting factors could inhibit fibrosis and inflammation and promote lesion repair.^13^ However, studies investigating the therapeutic effect of encapsulating both drugs and regenerative factors in multifunctional materials for the repair of lesion tissue are rare.

Inspired by the functions of MSCs primarily through the secretion of several factors and by the use of a promising biomimetic multifunctional nanoframework for targeted drug delivery, we herein designed and synthesized an MSC-inspired biomimetic multifunctional nanoframework (MBN) that is tailored to efficiently transport the growth-promoting factors secreted by MSCs, along with hydrophilic or hydrophobic drugs. In this work, two biomimetic nanoparticles, namely, rhein-encapsulated MBN (RMBN) and N-acetylcysteine (NAC)-encapsulated MBN (NMBN), were then designed and synthesized. These MBNs could relieve the acute symptoms of ALF through strong antioxidative and anti-inflammatory effects. The design of the MBN is summarized in Figure 1. In brief, freeze-dried MSC-conditioned media (MCM) and hydrophilic or hydrophobic drugs are encapsulated in poly(1,4-phenyleneacetone dimethylene thioketal) (PPADT), a ROS-sensitive polymer,^25,26^ and poly(vinyl alcohol) (PVA) during nanoparticle self-assembly.^13,26,27^ To increase the stability, the nanoparticles were then coated with the RBC membrane to yield the final product MBN. The presence of thioketal linkages in the polymer backbone of PPADT results in high resistance against enzymes and alkaline or acidic environments.^28^ Furthermore, PPADT can be readily cleaved into harmless acetone and thiol products in response to pathological levels of ROS *in vitro* or *in vivo*.^29^ Rhein (4,5-dihydroxyanthraquinone-2-carboxylic acid) is a lipophilic anthraquinone that is extensively found in medicinal herbs and has antioxidant activity.^30^ Rhein was chosen because its clinical application is limited by its poor water solubility and low bioavailability. Moreover, it has been reported to cause potential liver and kidney toxicity.^31−33^ N-Acetylcysteine (NAC) was chosen because it is a well-known hydrophilic antioxidant drug with a low bioavailability. The present study was designed to develop MBNs aimed at delivering growth-promoting factors secreted by MSCs, along with these hydrophilic or hydrophobic antioxidants with low bioavailability, to enhance the therapeutic effects of ALF. The effectiveness of these agents in prolonging drug circulation time as well as their hepaprotective effects on regenerating liver cells and promoting lesion repair was investigated in this study.

**Figure 1:**
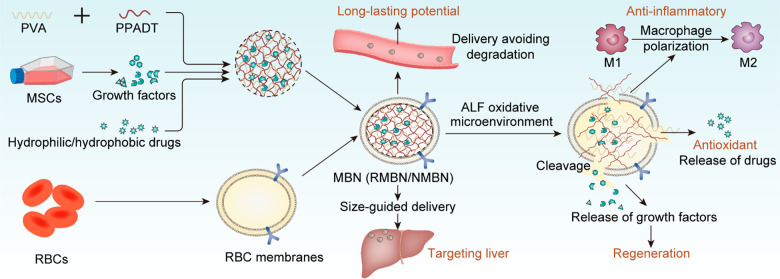
Design and multifunctional mechanism of an MSC-inspired biomimetic nanoframework for targeted treatment of ALF.

## Results

### The Fabrication and Characterization of RMBN

**Figure 2:**
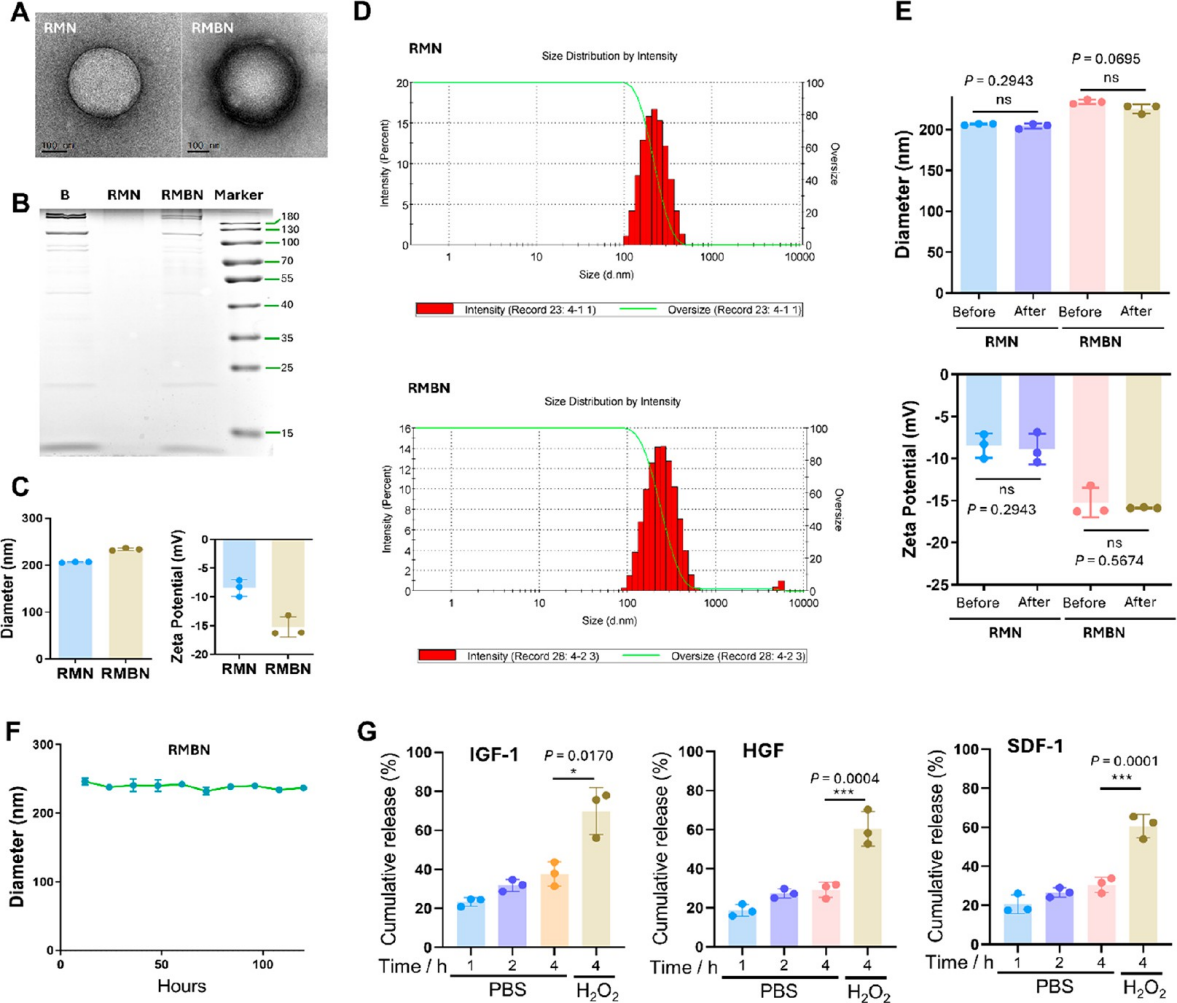
Characterization and biological properties of RMBN. (A) Typical TEM images of the RMN and RMBN. RMN: RMBN without the erythrocyte membrane coat. (B) Images of SDS-PAGE gels examining protein contents of RMN and RMBN. B: RBC membrane as a control group. (C) Diameters and zeta potential of RMN and RMBN (*n* = 3). (D) Size distributions of RMN and RMBN. (E) Diameters and zeta potential of RMBN before and after freeze/thaw (*n* = 3). (F) Size change of RMBN after storage at room temperature (*n* = 3). (G) Quantitative analyses on the releases of growth factors from RMBN over time with or without H_2_O_2_ stimulation. Data are expressed as means ± SD; *P < 0.05, **P < 0.01, ***P < 0.005, and ^ns^*P* > 0.05 compared with the vehicle group.

To validate the multifunctional mechanism of the MSC-inspired biomimetic nanoframework for the targeted treatment of ALF, we developed the hydrophobic drug-coated biomimetic nanoparticle RMBN using the hydrophobic anti-inflammatory and antioxidant active compound rhein.^30^ Rhein is isolated from rhubarb, which is one of the most important and ancient herbs in traditional Chinese medicine.^34^ In recent years, rhein has been widely used for the treatment of liver-related diseases in animal and clinical experiments.^35,36^ However, the applications of rhein have been largely limited by its poor solubility, low bioavailability, short half-life, easy degradation, and nonspecific organ toxicity.^37,38^ Morphological imaging confirmed by transmission electron microscopy (TEM) demonstrated that RBC membranes were coated with RMBN but not RMN, which is a control nanoparticle lacking an erythrocyte membrane coating (Figure 2A). Furthermore, the sodium dodecyl sulfate polyacrylamide gel electrophoresis (SDS-PAGE) results revealed similar protein compositions for the RMBN and RBC membranes, suggesting that the RMBNs were successfully coated with the RBC membrane (Figure 2B). Moreover, compared with RMN (Figure S1A), RMBN coated with erythrocyte membranes, which is crucial for prolonging the circulation time and improving the stability of nanomaterials in blood, has greater stability in serum. Particles displaying a slightly negative zeta potential are ideal for intravenous administration.^13^ Nanocharacterization using a Zetasizer revealed that the size of the nanoparticles changed from 206 nm (RMN) to approximately 234 nm (RMBN), and the zeta potential changed from −8.46 mV (RMN) to −15.32 mV (RMBN) after RBC membrane cloaking (Figure 2C,D), consistent with previously reported RBC nanoparticles.^13,39^ Notably, cryoconservation did not alter the zeta potential or size of the RMBN (Figure 2E). After long-term storage (120 h) and incubation in solutions with different pH values (pH 8.5, 7.4, 6.5, and 5.5), the RMBN was found to be stable and not significantly coaggregated (Figures 2F and S1B). According to the HPLC and bicinchoninic acid protein loading analysis results, the final biomimetic nanostructure had a loading capacity of 6.98% for rhein and 7.51% for freeze-dried MSC-conditioned medium, with loading efficacies of 63.89% and 52.40%, respectively. A RayBio Quantibody Cytokine protein array was used to characterize the secreted factors in the MSCs (Figure S2).^40^ A cytokine antibody array showed that MSC-secreted factors, such as insulin-like growth factor-1 (IGF-1), hepatocyte growth factor (HGF), stromal cell-derived factor-1 (SDF-1), transforming growth factor beta-1 (TGFb1), vascular endothelial growth factor (VEGF), VEGF-D, VEGF receptor-1 (VEGF R1), etc., were found at high levels. Moreover, release profiles of growth factors, such as IGF-1, HGF, and SDF-1, were detected after H_2_O_2_ exposure (Figure 2G). Compared with those of the vehicle, the growth factor release of the RMBN nanoparticles was significantly greater after 4 h of exposure to H_2_O_2_,^26^ which was further verified by the enhanced cumulative release of rhein from RMBN when the cells were exposed to 10 mM H_2_O_2_ (Figure S3). This finding indicated that RBC-coated RMBN could effectively promote the release of growth factors from RMBN in an ROS-responsive manner.

### Hepatoprotective Effects of RMBN in Lipopolysaccharide (LPS)/d-Galactosamine (D-GalN)-Induced ALF Murine Models

**Figure 3:**
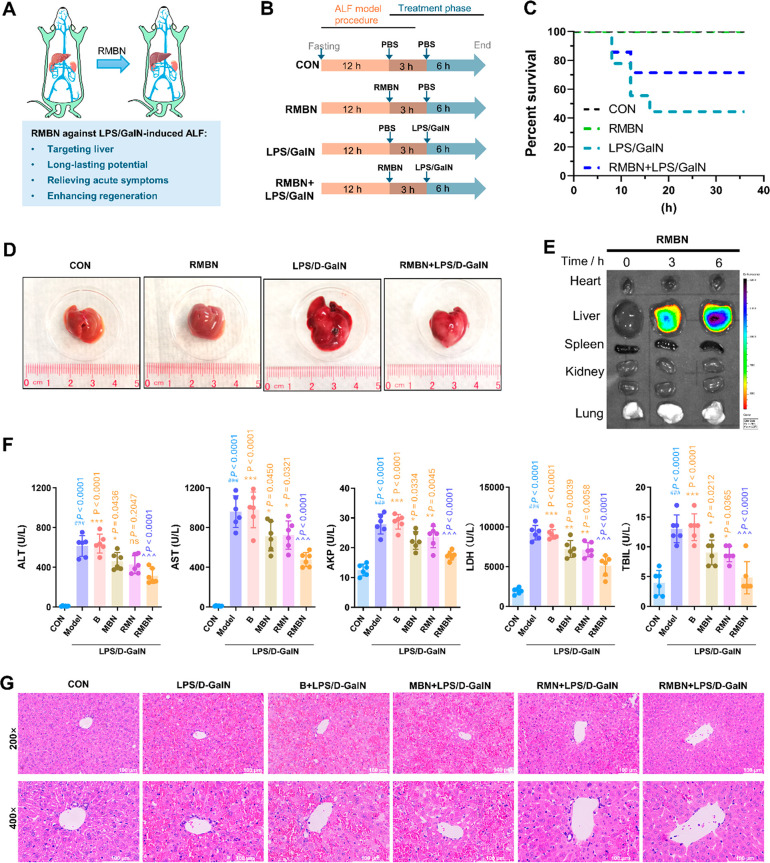
Hepatoprotective effects of RMBN in LPS/GalN-induced murine models of ALF. (A) Schematic showing the hepatoprotective effects of RMBN. (B) Schematic showing the ALF *in vivo* animal study design for evaluating the hepatoprotective effects of RMBN. (C) Survival rates of animal in various groups (*n* = 9). (D) Images of representative liver of mice after RMBN postinjection. (E) Biodistribution analysis of RMBN after postintravenous injections in mice with ALF. (F) Effects of MSC membrane (B), MBN, RMN, and RMBN on hepatology parameters, including serum ALT, AST, AKP, LDH, and TBIL. ^###^*P* < 0.005 (light blue), Model vs CON groups. *P < 0.05, **P < 0.01, ***P < 0.005, and ^ns^*P* > 0.05 (orange), compared with the RMBN group. ^∧∧∧^*P* < 0.005 (purple blue), RMBN vs Model groups. (G) H&E staining of representative liver of mice after the postinjection of B, MBN, RMN, and RMBN. Scale bar = 100 μm.

To assess the therapeutic potential of RMBN for reversing ALF in mice (Figure 3A), we used an LPS/GalN-induced mouse model of ALF (Figure 3B), which was further verified by evaluating the liver morphology (Figure 3C). After 36 h, intravenous RMBN therapy (blue) enhanced the survival rate of the LPS/GalN-induced ALF mice from 40% to 70% (Figure 3D). After intravenous injection, an increase in the level of RMBN was detected in the livers of the treated animals at 3 and 6 h (Figure 3E), suggesting effective liver retention of RMBN, which is an important property for quickly targeting the lesion site of ALF. Inspired by the positive effects of RMBN on survival in LPS/GalN-induced ALF mice, the hepatoprotective effects of RMBN on ALF were also investigated through the evaluation of liver function factors, ALT, aspartate aminotransferase (AST), alkaline phosphatase (AKP), lactate dehydrogenase (LDH), and total bilirubin (TBIL).^41^ As shown in Figures S4 and 3F, RMBN did not significantly alter liver function biomarkers; however, RMBN significantly reduced the increase in the serum ALT, AST, AKP, LDH, and TBIL levels induced by LPS/GalN. Compared to those of MSC membrane B, MBN, and RMN postinjection, the hepatoprotective effects of RMBN were greater according to the hepatology parameters in LPS/GalN-induced ALF murine models. These conclusions were further corroborated by H&E staining (Figure 3G). In addition, the combination of rhein and MCM effectively suppressed liver function factors compared with the individual combinations, although it may not be as effective as RMBN (Figure S5). These results demonstrated that RMBN has the potential to serve as a nontoxic drug in mice, as it does not cause hepatotoxicity. Collectively, RMBN exhibited hepatoprotective effects against LPS/GalN-induced toxicity in a murine model of ALF.

### Protective Effects of RMBN against Liver Oxidative Damage in LPS/GalN-Induced ALF Murine Models

**Figure 4:**
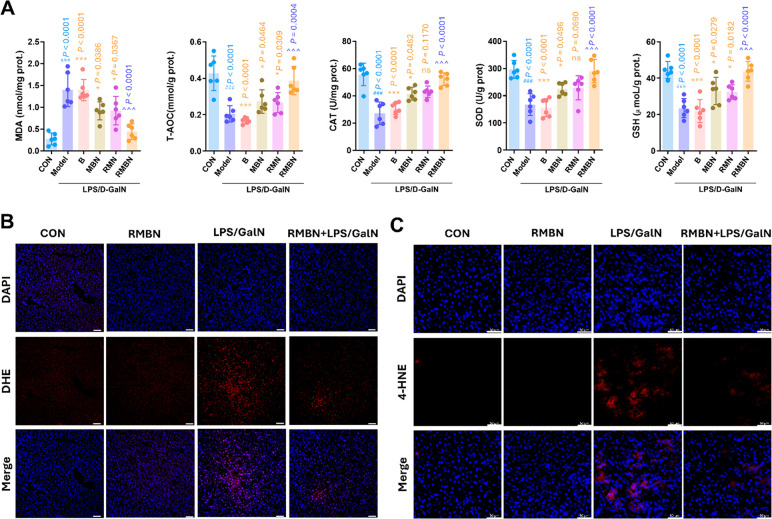
Protective effects of RMBN against liver oxidative damage in LPS/GalN-induced murine models of ALF. (A) Liver oxidative damage parameters, including MDA, SOD, CAT, T-AOC, and GSH, of liver were detected after MSC membrane B, MBN, RMN, and RMBN treatment. ^###^*P* < 0.005 (light blue), Model vs CON groups. *P < 0.05, **P < 0.01, ***P < 0.005, and ^ns^*P* > 0.05 (orange), compared with the RMBN group. ^∧∧∧^*P* < 0.005 (purple blue), RMBN vs Model groups. B: MSC membrane; MBN: RMBN without rhein; RMN: RMBN without erythrocyte membrane coat. (B) Representative DHE immunofluorescence in formalin-fixed liver sections taken from the mice of each group. (C) Representative 4-HNE immunofluorescence in formalin-fixed liver sections taken from the mice of each group. MDA, malondialdehyde; SOD, superoxide dismutase; CAT, catalase; T-AOC, total antioxidant capacity; GSH, glutathione. Scale bar = 50 μm.

In a subsequent study, the effects of RMBN on liver oxidative stress were determined. The oxidative damage parameters (e.g., SOD, catalase (CAT), total antioxidant capacity (T-AOC), and glutathione (GSH)) were markedly lower, whereas the level of the thiobarbituric acid reactive substance malondialdehyde (MDA) was greater in the livers of LPS/GalN-induced ALF mice than in those of control group (CON) mice (Figure 4A). Conversely, treatment with RMBN resulted in a significant increase in the levels of oxidative damage parameters and inhibited the level of MDA (Figure 4A). Moreover, the levels of intracellular ROS, as indicated by DHE immunofluorescence labeling,^42^ were significantly lower in the RMBN-treated mice than in the LPS/GalN-induced ALF model mice (Figure 4B). 4-Hydroxy-2-nonenal (4-HNE), a product of lipid peroxidation, is considered one of the most formidable reactive aldehydes and is identified as a biomarker of oxidative stress.^43^ In our study, we discovered that RMBN effectively suppressed the expression of 4-HNE (Figure 4C), which further confirmed the protective effects of RMBN against oxidative damage in the liver. In summary, these findings delineate the favorable impact of RMBN on LPS/GalN-induced hepatic oxidative damage in a murine model of ALF.

### RMBN Mitigated Hepatic Inflammation in LPS/GalN-Induced ALF Model Mice

**Figure 5:**
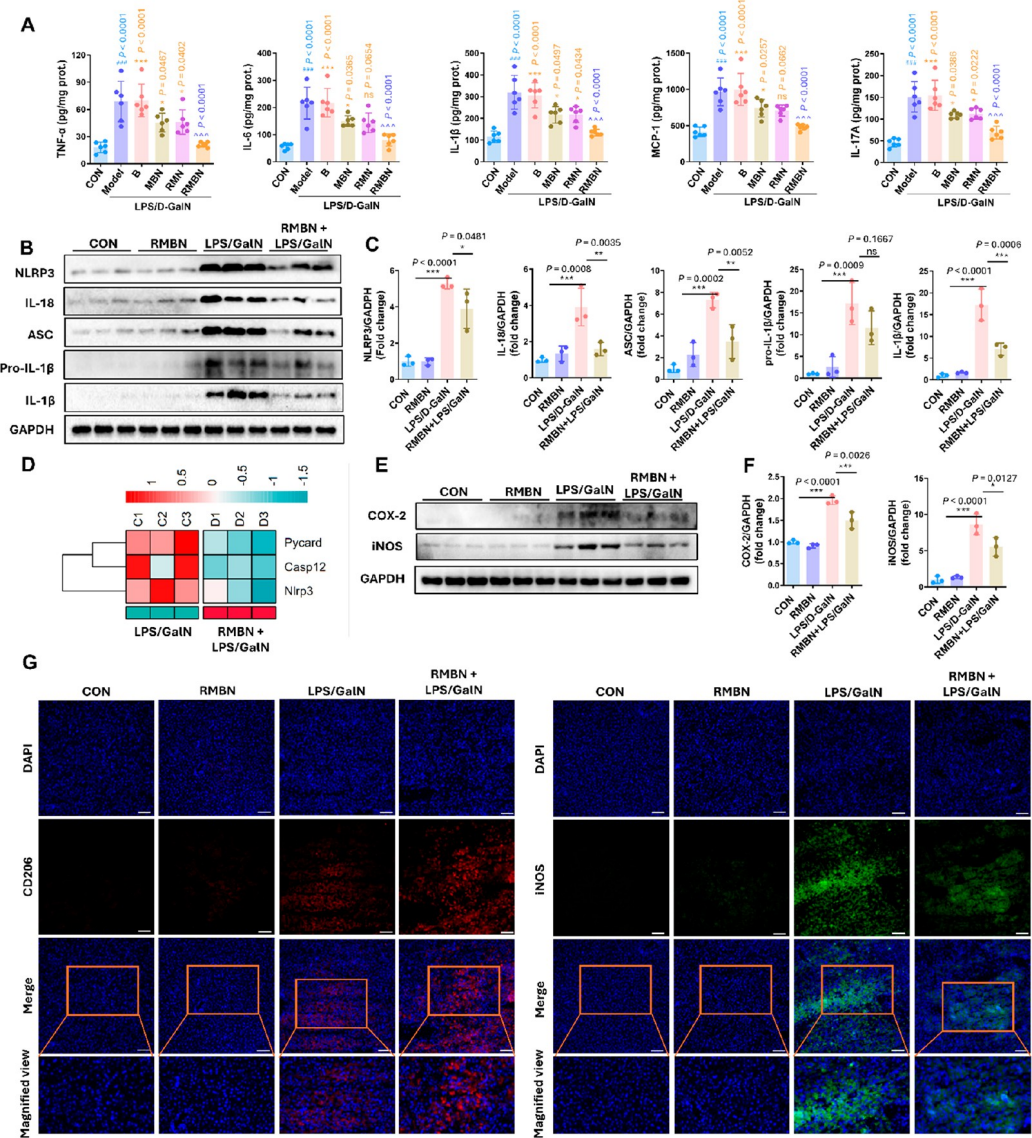
Effects of RMBN on hepatic inflammation in LPS/GalN-induced murine models of ALF. (A) Levels of TNF-α, IL-6, IL-1β, MCP-1, and IL-17A involved in liver inflammation were analyzed by ELISA in the liver (*n* = 7 mice). ^###^*P* < 0.005 (light blue), Model vs CON groups. *P < 0.05, **P < 0.01, ***P < 0.005, and ^ns^*P* > 0.05 (orange), compared with the RMBN group. ^∧∧∧^*P* < 0.005 (purple blue), RMBN vs Model groups. (B, C) Immunoblot analysis of factors in NLRP3-reated signaling pathways include NLRP3, IL-18, ASC, pro-IL-1β, and IL-1β in the liver. (D) Heatmap of the differential expressed genes related to the NLRP3 inflammasome activation in the livers of mimic ALF mice with or without the RMBN (*n* = 4). RMBN + LPS/GalN vs LPS/GalN groups. Colors represent log2-transformed count (log2) change after normalization. (E, F) Immunoblot analysis of factors in NLRP3-reated signaling pathways include NLRP3, IL-18, ASC, pro-IL-1β, and IL-1β in the liver. (G) Representative immunofluorescence staining of CD206^+^ (red) and iNOS (green) in mouse liver sections. Scale bar = 50 μm. Data are expressed as means ± SD (*n* = 3–7). *P < 0.05, **P < 0.01, ***P < 0.005, and ^ns^*P* > 0.05 compared with the vehicle group.

To ascertain the effects of RMBN on hepatic inflammation, the levels of proinflammatory cytokines, including TNF-α, IL-6, and monocyte chemoattractant protein-1 (MCP-1), were assessed using ELISA. As depicted in Figure 5A, the RMBN treatment resulted in a significant decrease in the IL-6 concentration and a decreasing trend in the TNF-α and MCP-1 levels in the LPS/GalN-induced ALF model mice, indicating that the RMBN treatment has the potential to alleviate the increase in the levels of proinflammatory cytokines. We also evaluated whether the RMBN-mediated alleviation of liver inflammation was associated with the NLRP3 and NF-κB pathways.^44^ The elevated levels of interleukin 17A (IL-17A) and IL-1β, which are related to the NLRP3 pathway, in ALF murine models were reversed by RMBN (Figure 5A). Notably, immunoblot analysis further demonstrated that RMBN reversed the increase in the protein expression of factors related to the NLRP3 pathway, including NLRP3, IL-18, apoptosis associated speck-like protein containing a caspase recruitment domain (ASC), pro-IL-1β, and IL-1β, in the liver tissue of LPS/GalN-induced ALF model mice (Figure 5B,C). Furthermore, these findings were also corroborated by the reduced transcription levels of genes associated with NLRP3 inflammasome activation in our RNA-seq data set, particularly the downregulated gene *NLRP3* (Figure 5D). Inflammation is strongly associated with cyclooxygenase-2 (COX-2) and inducible nitric oxide synthase (iNOS).^45^ Therefore, we investigated the effect of RMBN on COX-2 and iNOS in the liver tissue of ALF model mice and found that RMBN substantially inhibited the expression of COX-2 and iNOS in LPS/GalN-induced ALF model mice (Figure 5E,F). Moreover, we found that the expression of CD206^+^, which is an M2-type macrophage marker, was moderately increased in the liver tissue of the model mice compared to that in the liver tissue of the ALF model mice. In contrast, the levels of M1-type macrophage-derived proinflammatory cytokines, such as iNOS, were significantly decreased (Figure 5G). The M2 macrophage differentiation effect of RMBN was further verified by flow cytometry analysis of the macrophage-derived cytokines CD86^+^ and CD206^+^ (Figure S6). These data showed that RMBN strongly promoted the polarization of macrophages toward the M2 phenotype after RMBN treatment in the LPS/GalN-induced ALF murine models.

### RMBN Enhances Hepatic Regeneration in LPS/GalN-Induced ALF Murine Models

**Figure 6:**
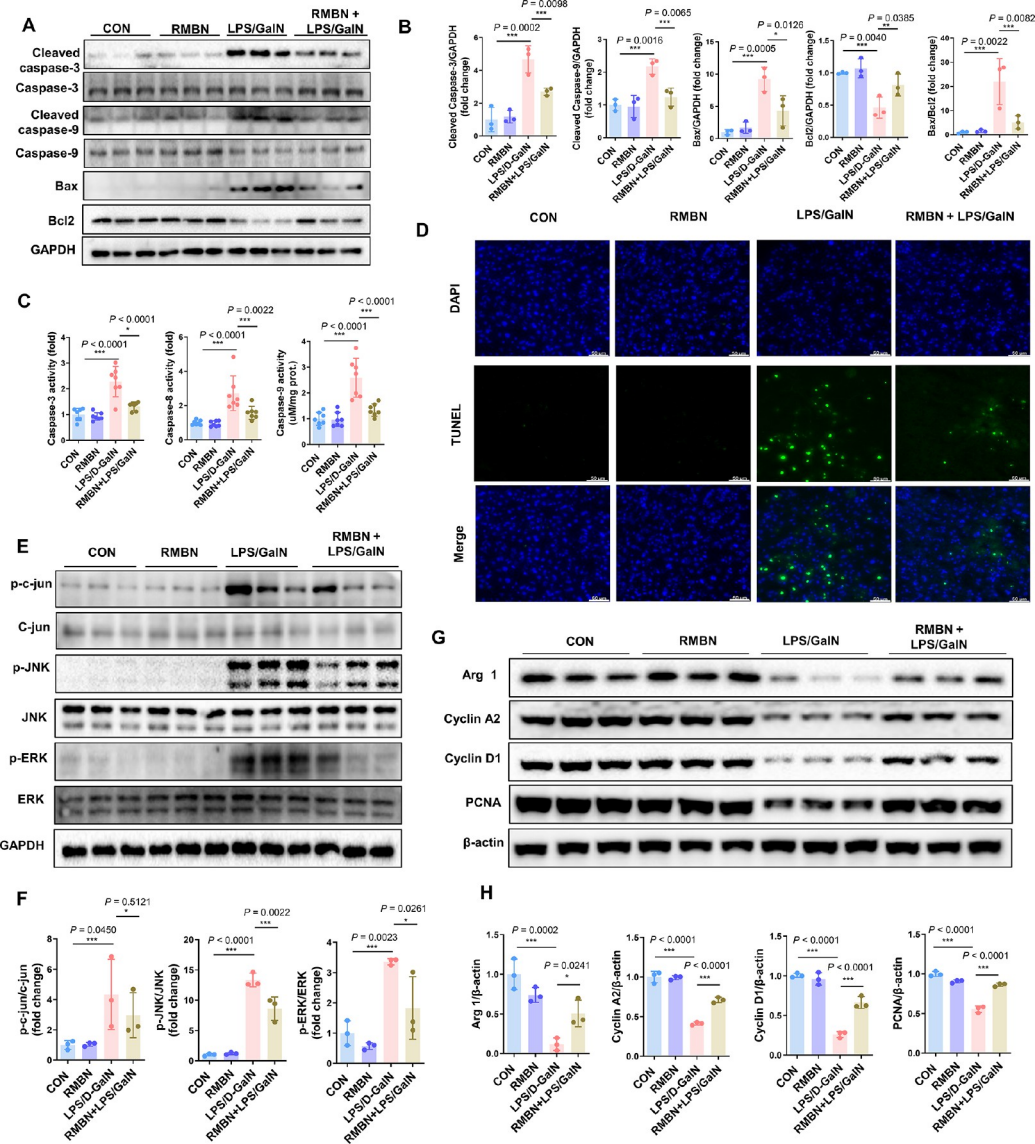
Effects of RMBN on hepatic regeneration in the LPS/GalN-induced murine model of ALF. (A) Immunoblot analysis of apoptotic proteins, including cleaved caspase-3, caspase-3, cleaved caspase-9, caspase-9, Bax, and Bcl-2, in the liver of LPS/GalN-induced mimic ALF mice. (B) Densitometry analysis of apoptotic protein levels on the Western blot. (C) Activity analysis of apoptotic proteins, including caspase-3, caspase-8, and caspase-9, using a commercial kit. (D) Representative immunofluorescence staining of TUNEL (green) in mouse liver sections. Scale bar = 50 μm. (E) Immunoblot analysis of MAPK family members, including C-jun, JNK, and ERK, in the liver of LPS/GalN-induced mimic ALF mice. (F) Densitometry analysis of apoptotic protein levels on the Western blot. (G) Immunoblot analysis of cell cycle progression and proliferation, including Arg 1, Cyclin A2, Cyclin D1, and PCNA, in the liver of LPS/GalN-induced mimic ALF mice. (H) Densitometry analysis of these apoptotic protein levels on the Western blot. Data are expressed as means ± SD (*n* = 3–7). *P < 0.05, **P < 0.01, ***P < 0.005, and ^ns^*P* > 0.05 compared with the vehicle group.

To verify the effects of RMBN on liver regeneration, the levels of apoptotic proteins, including cleaved caspase-3, cleaved caspase-3, cleaved caspase-9, caspase-9, Bcl-2, and Bcl-2 antagonist X (Bax), were measured. As shown in Figure 6A,B, in ALF model mice, the RMBN treatment significantly decreased the levels of pro-apoptotic proteins, including cleaved caspase-3, cleaved caspase-9, and Bax. Conversely, the levels of the antiapoptotic protein Bcl-2 were significantly enhanced by RMBN. Additionally, the increase in the Bax/Bcl-2 ratio in the livers of ALF mice was markedly suppressed by RMBN administration. Apart from the downregulated apoptotic protein levels, the hepatoprotective effect of RMBN was further substantiated by the diminished activity of apoptotic proteins, including caspase-3, caspase-8, and caspase-9, as determined by using commercial kits (Figure 6C). Furthermore, terminal deoxynucleotidyl transferase-mediated dUTP nick end labeling (TUNEL) staining revealed that apoptosis in LPS/GalN-induced ALF was effectively reduced by RMBN injection (Figure 6D). The mitogen-activated protein kinase (MAPK) family members, including Jun N-terminal kinases (JNK), extracellular signal-regulated kinase (ERK), and C-jun, are important proteins for cellular responses to oxidative stress, as well as for the regulation of cell differentiation, proliferation, and apoptosis.^46,47^ Previous studies have shown that suppressing the protein expression of phosphorylated MAPK family members enables the inhibition of apoptosis induced by LPS/GalN.^48^ In this work, upon treating the livers of ALF mice with RMBN, the LPS/GalN-induced upregulation of phosphorylated C-jun, JNK, and ERK was attenuated (Figure 6E,F). These results suggest that inhibiting the MAPK pathway to reduce hepatocyte apoptosis may contribute to the protective effect of RMBN against LPS/GalN-induced liver injury. Additionally, in the liver of LPS/GalN-induced ALF model mice, RMBN significantly increased the expression of proteins related to cell cycle progression and proliferation factors, including Arginase 1 (Arg 1), Cyclin A2, Cyclin D1, and proliferating cell nuclear antigen (PCNA) (Figure 6G,H). These data further indicated that RMBN could diminish liver apoptosis and promote liver regeneration in ALF murine models.

### RMBN Ameliorates the ALF Response in an LPS/GalN-Induced ALF Murine Model

**Figure 7:**
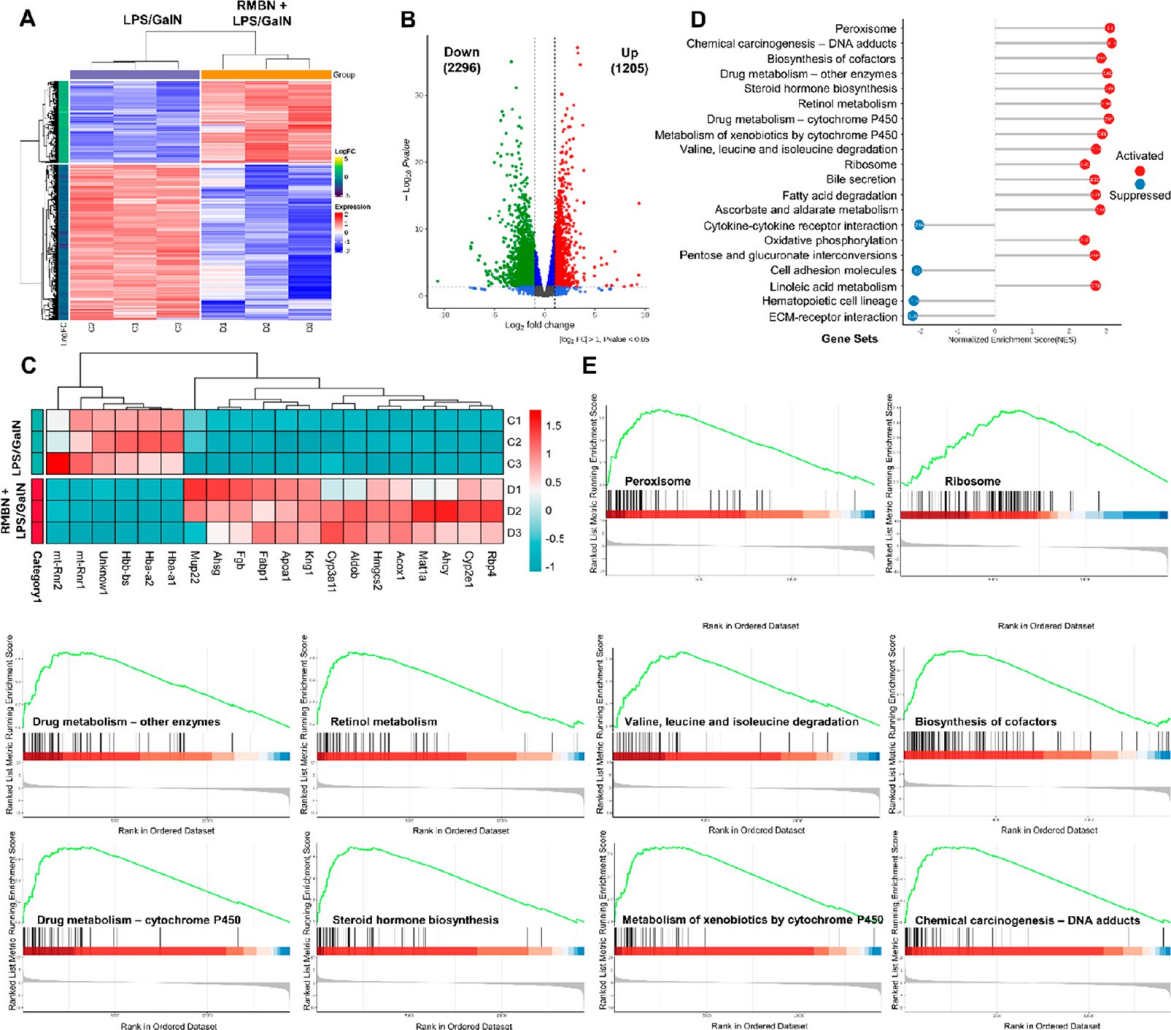
RMBN ameliorates the ALF response in LPS/GalN-induced murine models of ALF. (A) Cluster analysis of the differential expressed genes. RMBN (RMBN + LPS/GalN) vs vehicle (LPS/GalN) group. (B) Volcano plot of the differential expressed genes. RMBN vs vehicle group. (C) Heatmap of the top 20 differential expressed genes. RMBN vs vehicle group. (D) Enrichment analyses of the differential expressed genes using GO analysis. RMBN vs vehicle group. (E) Gene set enrichment analysis (GSEA) of the differential expressed genes of representative pathways in the liver of mimic ALF mice. RMBN vs vehicle group.

Next, we employed RNA-seq analysis to assess the hepatoprotective mechanism of RMBN in ALF murine models. In the presence of RMBN, 3501 differentially expressed genes (DEGs) were identified, comprising 1205 upregulated genes and 2296 downregulated genes (Figure 7A,B). The top 20 DEGs, which include mt-Rnr2, Cyp2e1, and Apoa1, are shown in Figure 7C. Gene set enrichment analysis (GSEA) and Gene ontology (GO) enrichment analysis were subsequently performed to elucidate the potential underlying mechanism involved. According to the enrichment analysis, RMBN might downregulate immune/inflammatory biological processes and pathways, including cytokine–cytokine receptor interactions, cell adhesion molecules, hematopoietic cell lineages, and extracellular matrix–receptor interactions, and upregulate liver regeneration-related biological processes and pathways, such as peroxisomes and biosynthesis of cofactors, to ameliorate the ALF response in murine models (Figure 7D,E). In brief, RMBN ameliorated the ALF response in LPS/GalN-induced ALF murine models, potentially through the downregulation of immune/inflammatory processes and the upregulation of liver regeneration-related biological processes and pathways, which aligns with the aforementioned results.

### NMBN Exhibited Hepatoprotective Effects against Acetaminophen (APAP)-Induced ALF

**Figure 8:**
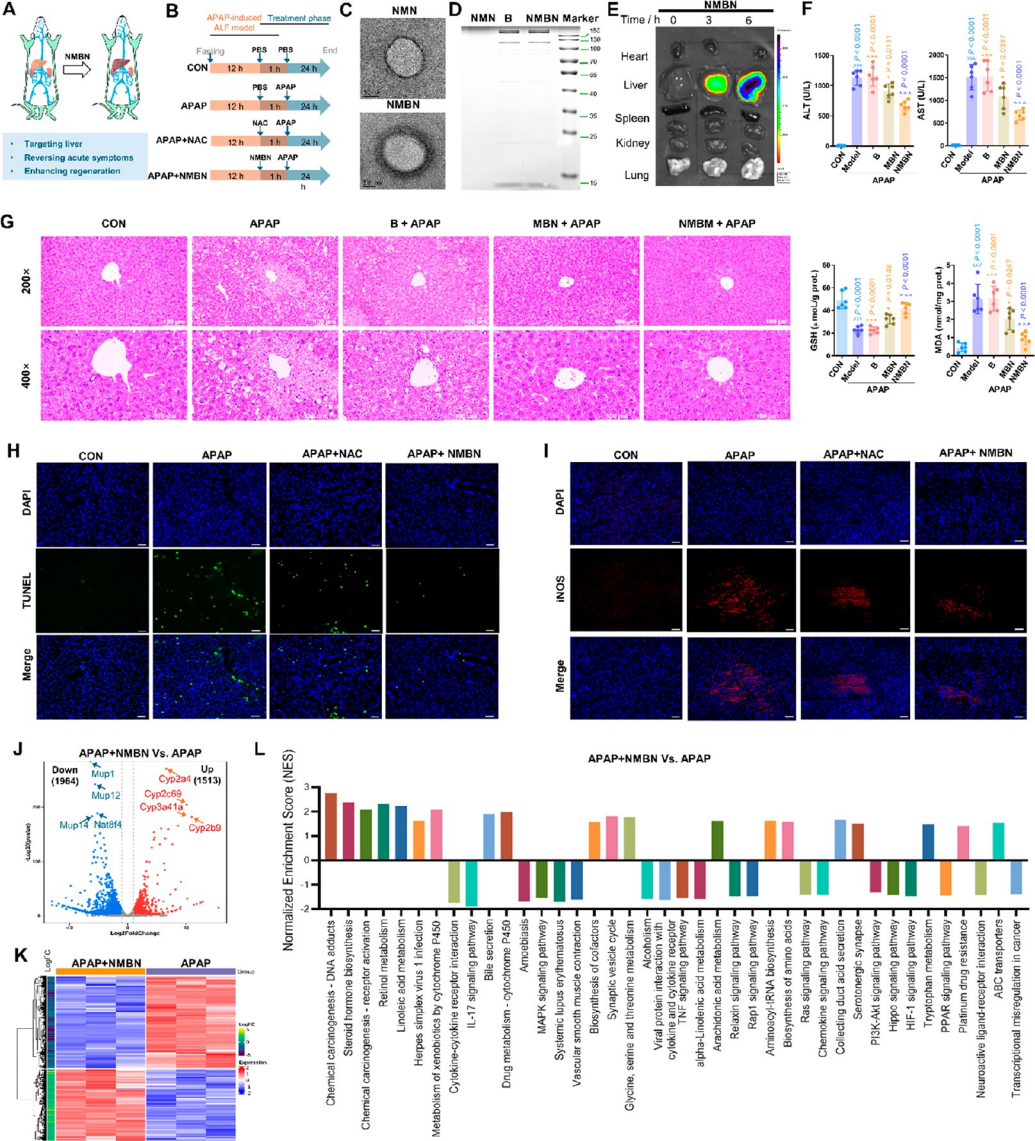
Design and characterization of NMBN for the treatment of the APAP-induced murine model of ALF. (A) Schematic showing the hepatoprotective effects of NMBN. (B) Schematic showing the ALF *in vivo* animal study design for evaluating the hepatoprotective effects of NMBN. (C) Typical TEM images of NMN and NMBN. (D) Images of SDS-PAGE gels examining protein contents of NMBN. B: RBC membrane as control group. (E) Biodistribution analysis of NMBN after postintravenous injections in mice with ALF. (F) Effects of B, MBN, and NMBN on hepatology parameters. B: MSC membrane; MBN: NMBN without NAC. (G) Histological H&E staining of mice live after the postinjection of B, MBN, and NMBN. Scale bar = 100 μm. Data are as means ± SD (*n* = 6). ^###^*P* < 0.005 (light blue), Model vs CON groups. *P < 0.05, **P < 0.01, ***P < 0.005, and ^ns^*P* > 0.05 (orange), compared with the NMBN group. ^∧∧∧^*P* < 0.005 (purple blue), NMBN vs Model groups. (H, I) Representative immunofluorescence staining of TUNEL (green) and iNOS (red) in mouse liver sections. Scale bar = 50 μm. (J) Volcano plot of the differential expressed genes. NMBN (APAP + NMBN) vs vehicle (APAP) group. (K) Cluster analysis of the differential expressed genes. NMBN vs vehicle group. (L) Enrichment analyses of the differential expressed genes using GO analysis. NMBN vs vehicle group.

APAP overdose-induced hepatotoxicity is the most prevalent cause of ALF, and treatment options other than NAC are limited.^49^ Inspired by the hepatoprotective effects of the hydrophobic rhein in combination with RMBN, the effects of NMBN, a biomimetic nanoparticle synthesized from ALF using hydrophilic NAC with a loading capacity of 9.72% and loading efficacy of 59.72%, were also investigated by evaluating factors related to liver injury in APAP-induced ALF murine models (Figure 8A,B). The morphology confirmed by TEM imaging demonstrated that the RBC membrane was coated on NMBNs but not on the control nanoparticle NMN, which is a control nanoparticle lacking an erythrocyte membrane coating (Figure 8C), which was also further confirmed by SDS-PAGE imaging (Figure 8D). The combination effect of NAC and MCM was initially assessed by using hepatology parameter analysis. The results showed that the combination had a promising hepatoprotective effect on APAP-induced ALF (Figure S7). Like in the case of RMBN, NMBN could effectively target livers, as evidenced by the increase in the signal density in the livers at 3 and 6 h postintravenous injection (Figure 8E). Moreover, compared with MBN, NMBN exhibited greater hepatoprotective effects against APAP-induced ALF, as evidenced by a reduction in the increase in the serum ALT, AST, and MDA levels and an increase in the serum GSH level in an APAP-induced ALF murine model (Figure 8F), which was further confirmed by H&E staining (Figure 8G). Additionally, TUNEL and iNOS staining revealed that NMBN injection effectively reduced apoptosis in APAP-induced ALF, which could be, at least in part, regulated by promoting macrophage polarization from the M1- to the M2-type (Figure 8H,I). RNA-seq analysis further revealed 3477 DEGs in the presence of NMBN, including 1513 upregulated genes and 1964 downregulated genes, in APAP-induced ALF murine models (Figure 8J,K). According to the enrichment analysis, NMBN could effectively reverse APAP-induced changes in gene expression and corresponding biological processes and pathways (Figures 8L and S8), including the IL-17 signaling pathway, cytokine–cytokine receptor interaction, retinol metabolism, bile secretion, chemical carcinogenesis, steroid hormone biosynthesis, chemical carcinogenesis-receptor activation, linoleic acid metabolism, etc., to ameliorate the ALF response in APAP-induced ALF murine models (Figures S9 and S10). In brief, NMBN ameliorates the ALF response in APAP-induced ALF mice potentially by downregulating immune/inflammatory processes and upregulating liver regeneration pathways, which is consistent with the aforementioned results.

## Discussion

ALF, also known as fulminant hepatic failure, is a rare yet serious clinical syndrome characterized by acute hepatocyte dysfunction, widespread hepatocellular necrosis, and subsequent multiorgan dysfunction.^50^ ALF is usually caused by various etiologies, including viral and bacterial diseases, poisonous substances, and other autoimmune or hereditary diseases.^51^ Liver transplantation has been recognized as a long-term effective treatment option for ALF.^52^ However, this approach is limited by the scarcity of donor organs, the risk of immune rejection, and high medical costs.^52^ Therefore, alternative treatments and regenerative strategies are urgently needed for ALF.

ROS accumulation is a critical pathological characteristic closely linked to ALF development and progression. ROS buildup leads to hepatocyte damage and necrosis, causing the release of ALT from damaged cells into the bloodstream.^15^ Clinical and experimental evidence has demonstrated that antioxidant compounds, both hydrophobic and hydrophilic, exhibit promising effects on liver injury.^53^ In animal and clinical studies, the antioxidant compound rhein has been widely used to treat liver-related diseases.^54^ However, hydrophobic rhein typically has poor solubility, is easily degraded, has a short half-life, has low bioavailability, or has nonspecific organ toxicity.^37,38^ Moreover, as an active antioxidant, rhein has been reported to have a protective effect on the liver, while other studies have shown that rhein has potential hepatorenal toxicity, indicating that rhein has bidirectional regulatory effects on liver disease.^55^ Currently, hydrophilic NAC is the only approved drug for treating APAP-induced liver injury. However, treatment of ALF with NAC, whether through oral or intravenous administration, can be hindered by severe adverse events.^56^ In this study, we designed an oxidative microenvironment-responsive biomimetic nanoframework, MBN, for reversing ALF. As a proof-of-concept, we synthesized two biomimetic nanoparticles using this framework: RMBN, which contains hydrophobic rhein, and NMBN, which contains hydrophilic NAC. Both RMBN and NMBN exhibited promising antioxidative effects against liver oxidative damage in LPS/GalN-induced and APAP-induced ALF mouse models, respectively.

Apart from the antioxidant effect, comprehensive treatment solutions, such as alleviating acute symptoms and promoting lesion repair, are also necessary for ALF treatment. In the present study, the fabricated biomimetic RMBN not only reduced liver oxidative damage but also exhibited strong hepatoprotective effects by decreasing liver inflammation and enhancing liver regeneration in mice with LPS/GalN-induced ALF. This observation is consistent with the RNA-seq analysis results. Moreover, MSC therapy shows great promise as a strategy for liver regeneration. Preclinical and clinical studies have demonstrated the efficacy of regenerative factors for treating liver diseases, such as liver fibrosis, cirrhosis, and liver failure. Unlike MRIN, which encapsulates the regenerative factors of MSCs,^13^ RMBN and NMBN are promising ALF treatments through the regulation of rapid release of drugs and growth factors, as well as the ability to alleviate acute symptoms and enhance liver regeneration.

Furthermore, both RMBN and NMBN biomimetic nanoparticles exhibited promising liver-targeting abilities. In recent years, erythrocyte membrane-coated nanoparticles have shown great success in achieving long-term blood circulation. Both the RMBN and NMBN were coated with erythrocyte membranes, which retained crucial membrane protein complexes and enhanced the blood stability of the nanoparticles. Additionally, these nanoparticles were approximately 200 nm in size, providing a robust element for organ retention in the liver.^13,57^ In our study, after intravenous delivery, biomimetic particles based on the nanoframework were observed to rapidly accumulate in the liver of ALF model mice within 3 h, which is crucial for relieving acute symptoms. In brief, the nanoframework MBN offers an innovative and readily available approach for treating liver failure.

## Conclusions

In this study, we successfully prepared an oxidative microenvironment-responsive biomimetic nanoframework, MBN, for reversing ALF. The nanoframework, which carries both hydrophobic/hydrophilic drugs and regenerative factors from MSCs, was further coated with RBC membranes to improve its stability in blood. Using this nanoframework, we synthesized two biomimetic nanoparticles, RMBN and NMBN, encapsulating hydrophobic rhein and hydrophilic NAC, respectively. Our study demonstrated that, by reducing liver oxidative damage and inflammation and enhancing liver regeneration in mice with LPS/GalN-induced ALF, RMBN exhibited strong hepatoprotective effects. RNA-seq analysis further revealed that RMBN ameliorates the ALF response in LPS/GalN-induced ALF mice potentially by downregulating immune-inflammatory processes and upregulating liver regeneration pathways. In addition to the use of hydrophobic drugs encapsulating MBN for reversing LPS/GalN-induced ALF, our study also fabricated biomimetic nanoparticles encapsulating the hydrophilic drug NAC to evaluate their hepatoprotective effects against APAP-induced ALF. Overall, the biomimetic nanoframework MBN, which has long-term potential, exhibits promising hepatoprotective activity for the effective delivery of growth-promoting factors secreted by MSCs, as does the combination of hydrophilic rhein or hydrophobic NAC, which effectively accelerates the targeting of liver lesions, alleviates acute symptoms through robust antioxidative and anti-inflammatory effects, and promotes hepatic regeneration. However, it is important to note that the LPS/GalN- and APAP-induced models used in this study have their own limitations and cannot fully mimic human ALF. However, further research is still needed to validate the use of the MBN as an alternative therapeutic and regenerative strategy for effectively relieving ALF or even replacing the pressure of orthotopic liver transplantation for ALF in clinical practice.

## Experimental Section

### Materials

Rhein was purchased from Aladdin Industrial Corporation Co., Ltd. (Shanghai, China). NAC and APAP were purchased from Sigma-Aldrich Chemical Co. (St. Louis, MO, USA). Mouse CXCL12 ELISA Kit, Mouse IGF-1 ELISA Kit, and Mouse HGF ELISA Kit were purchased from Elabscience Biotechnology Co., Ltd. (Wuhan, China).

### Fabrication and Characterization of RMBN and NMBN

MSC conditioned media-loaded PPADT nanoparticles (MSC-NPs) were first fabricated as previously described.^13^ In brief, the drugs rhein and NAC were initially dissolved in organic or aqueous phases. The entire content was then sonicated on ice for emulsification. Following the secondary emulsion, the entire content was stirred overnight to facilitate solvent evaporation. To coat RBC vesicles with MSC-NPs, 0.5 mL of each was mixed and extruded 11 times. The resulting RMBN and NMBN were then centrifuged at 800*g* to remove the excess membrane debris. The size and surface charge of the nanoparticles were analyzed using NanoSight (Malvern, UK). TEM was used to examine the morphologies of RMBN and NMBN, which were imaged after negative staining with 1 wt % uranyl acetate. SDS-PAGE was utilized to determine whether RMBN and NMBN had signature proteins similar to those of RBCs by revealing the protein components of RBC membrane vesicles.

### Murine Models of ALF Induced by LPS/GalN and RMBN Treatment

Male C57BL/6J mice (8–10 weeks old, 18–20 g) were procured from the Animal Research Core at the University of Macau. All animal experiments received approval from the Animal Research Ethics Committee at the Institute of Chinese Medical Sciences, University of Macau. Mice were housed in an individually ventilated cage (IVC) system with regulated temperature and light conditions. Following a week-long acclimatization period, mice were randomly assigned to four groups: Control (CON) group, RMBN group, LPS/GalN group, and RMBN + LPS/GalN group. The RMBN group and RMBN + LPS/GalN mice received RMBN (4 mg/kg) via tail vein injection for 3 h, followed by the RMBN + LPS/GalN group and GalN/LPS group being treated with 10 μg/kg LPS and 500 mg/kg GalN (Sigma) dissolved in phosphate-buffered saline.^34^ Mice were euthanized 6 h post LPS/GalN injection, with blood samples and liver tissue collected for subsequent analysis, in accordance with the manufacturer’s protocols. For survival rates and biodistribution analysis of RMBN, mice were euthanized after 0, 3, 6, or 36 h postintravenous injection.

### Reactive Oxygen Species Assessment

Hepatic ROS levels were evaluated using dihydroethidium ethylenide (DHE) (Beyotime, Nanjing, China). Cryostat sections of freshly acquired liver samples were incubated with DHE at 37 °C in light-protected conditions for 30 min. Nuclear staining was performed using DAPI for 10 min. For 4-HNE determination, liver sections were incubated with the 4-HNE antibody and Alexa Fluor 594-conjugated secondary antibody. Fluorescent signaling was recorded and analyzed by using a confocal imaging system (Olympus, Tokyo, Japan).

### RNA Sequencing Analysis

Fresh mouse liver tissue exposed to either vehicle or treatment was collected, and the total RNA was extracted using an RNA isolation kit. To ensure high-quality clean reads, raw data from the Illumina PE150 platform were trimmed. Enrichment analysis was conducted on the gene ontology (GO) and Kyoto Encyclopedia of Genes and Genomes (KEGG) databases, following the methodology established by Guangzhou IGE Biotechnology Ltd. (Guangzhou, China). Gene set enrichment analysis (GSEA) was also performed to study gene enrichment in specific functions across all expressed genes.

### Statistical Analysis

Data were presented as means ± standard deviation (SD). Tukey’s post hoc test and one-way analysis of variance (ANOVA) were used to measure the difference among three or more comparison groups using GraphPad Prism 6.0 software. Differences between groups were considered statistically significant at a *P*-value of < 0.05.

## Supporting Information Available

Stability of the RMBN in serum, alkaline, and acidic environments; characterization of representative MSC-secreted factors by protein array; H_2_O_2_ responsive drug release of RMBN; effects of RMBN on hepatology parameters, including serum ALT, AST, AKP, LDH, and TBIL; synergistic effect of the combination of Rhein and MSC-conditioned media in LPS/GalN-induced murine models of ALF; effect of RMBN on M1 and M2 macrophage differentiation; synergistic effect of the combination of NAC and MCM in APAP-induced mimic ALF; characterization of APAP-induced mimic ALF; GSEA of the differential expressed genes of APAP vs control group; GSEA of the differential expressed genes of NMBN + APAP vs APAP group (PDF)

## Abbreviations

4-HNE: 4-hydroxynonenal
ALF: acute liver failure
ALT: alanine transaminase
AKP: alkaline phosphatase
ANOVA: analysis of variance
APAP: acetaminophen
Arg 1: arginase 1
ASC: apoptosis associated speck-like protein containing a caspase recruitment domain
AST: aspartate aminotransferase
Bax: Bcl-2 antagonist X
CAT: catalase
DEGs: differentially expressed genes
COX-2: cyclooxygenase-2
DHE: dihydroethidium
D-GalN: d-galactosamine
ERK: extracellular signal-regulated kinase
GO: gene ontology
GSEA: gene set enrichment analysis
HGF: hepatocyte growth factor
IGF-1: insulin-like growth factor-1
IL-1β: interleukin 1β
IL-6: interleukin 6
IL-17A: interleukin 17A
iNOS: inducible nitric oxide synthase
GAPDH: glyceraldehyde-3-phosphate dehydrogenase
GSH: glutathione
JNK: Jun N-terminal kinase
LDH: lactate dehydrogenase
LPS: lipopolysaccharide
MAPK: mitogen-activated protein kinase
MBN: MSC-inspired biomimetic multifunctional nanoframework
MCP-1: monocyte chemoattractant protein-1
MDA: malondialdehyde
MCM: MSC-conditioned media
MSCs: mesenchymal stem cells
MSC-NPs: MSC conditioned media-loaded PPADT nanoparticles
NAC: N-acetylcysteine
NMBN: N-acetylcysteine (NAC)-encapsulated MBN
PCNA: proliferating cell nuclear antigen
PLGA: poly lactic-*co*-glycolic acid
PPADT: poly(1,4-phenyleneacetone dimethylene thioketal
PVA: poly(vinyl alcohol)
RBC: red blood cell
RMBN: rhein-encapsulated MBN
RMN: RMBN without erythrocyte membrane coat
ROS: reactive oxygen species
SD: standard deviation
SDF-1: stromal cell-derived factor-1
SDS-PAGE: sodium dodecyl-sulfate polyacrylamide gel electrophoresis
SOD: superoxide dismutase
T-AOC: total antioxidant capacity
TBIL: total bilirubin
TEM: transmission electron microscopy
TGF-β1: transforming growth factor-beta 1
TNF-α: tumor necrosis factor-α
TUNEL: terminal deoxynucleotidyl transferase-mediated dUTP nick end labeling
VEGF: vascular endothelial growth factor
VEGF R1: VEGF receptor-1
