# Supplementary material for: A Biomimetic Multifunctional Nanoframework for Symptom Relief and Restorative Treatment of Acute Liver Failure
Source: ACS Nano. 2024 Jan 31;18(7):5951–64. doi: 10.1021/acsnano.4c00173 (PMC10883031; doi:10.1021/acsnano.4c00173)
Supplement: Supplementary file 1 — nn4c00173_si_009.pdf [file nn4c00173_si_009.pdf]

Supplementary Materials

A Biomimetic Multifunctional Nanoframework for Symptom Relief and Restorative Treatment of Acute Liver Failure

Ruibing Feng,# Yu Fan,# Xinya Zhang,# Lanmei Chen, Zhang-Feng Zhong, Yitao Wang,* Hua Yu,* Qing-Wen Zhang,* and Guodong Li*

Corresponding Author

Guodong Li − *Macao Centre for Research and Development in Chinese Medicine, State Key Laboratory of Quality Research in Chinese Medicine, Institute of Chinese Medical Sciences, University of Macau, Macao SAR 999078, P.R. China; Zhuhai UM Science and Technology Research Institute, Zhuhai 519031, P.R. China*; Email: guodongli@um.edu.mo

Qing-Wen Zhang – *State Key Laboratory of Quality Research in Chinese Medicine, Institute of Chinese Medical Sciences, University of Macau, Macao SAR 999078, P.R. China*; Email: qwzhang@um.edu.mo

Hua Yu − *State Key Laboratory of Quality Research in Chinese Medicine, Institute of Chinese Medical Sciences, University of Macau, Macao SAR 999078, P.R. China*; Email: bcalecyu@um.edu.mo

Yitao Wang − *Macao Centre for Research and Development in Chinese Medicine, State Key Laboratory of Quality Research in Chinese Medicine, Institute of Chinese Medical Sciences, University of Macau, Macao SAR 999078, P.R. China*; Email: ytwang@um.edu.mo

Authors

Ruibing Feng − *State Key Laboratory of Quality Research in Chinese Medicine, Institute of Chinese Medical Sciences, University of Macau, Macao SAR 999078, P.R. China.*

Yu Fan − *Macao Centre for Research and Development in Chinese Medicine, State Key Laboratory of Quality Research in Chinese Medicine, Institute of Chinese Medical Sciences, University of Macau, Macao SAR 999078, China; Zhuhai UM Science and Technology Research Institute, Zhuhai 519031, P.R. China.*

Xinya Zhang − *State Key Laboratory of Quality Research in Chinese Medicine, Institute of Chinese Medical Sciences, University of Macau, Macao SAR 999078, China; Zhuhai UM Science and Technology Research Institute, Zhuhai 519031, P.R. China.*

Lanmei Chen − *The Marine Biomedical Research Institute of Guangdong Zhanjiang, School of Ocean and Tropical Medicine, Guangdong Medical University, Zhanjiang, Guangdong, 524023, P.R. China.*

Zhang-Feng Zhong − *Macao Centre for Research and Development in Chinese Medicine, State Key Laboratory of Quality Research in Chinese Medicine, Institute of Chinese Medical Sciences, University of Macau, Macao SAR 999078, P.R. China.*

Supplementary Methods

1.1 Generation of RBC membrane vesicles

RBC shells were firstly synthesized as previously described.[1] To prepare RBC membrane vesicles, RBC shells were subjected to three freeze/thaw cycles. After that, the collected RBC shells were extruded through membranes with pore sizes of 400 and 200 nm using an extruder (Avanti Polar Lipids).

1.2 Loading efficiency and capacity into the biomimetic nano-framework

The amount of drug incorporated into the biomimetic nano-framework was quantified using a high-performance liquid chromatography (HPLC) analysis after the release of the encapsulated drugs as previously described.[2] MSC-conditioned medium protein loading was evaluated by the nanoparticle digestion method as previously described.[3] Loading efficiency and loading capacity (LC) of all samples were determined using the formulas:

Loading efficiency = (weight of drug incorporated / weight of drug added during the formulation) × 100 %;

Loading capacity (LC) = (weight of drug incorporated / weight of drug-loaded nanoparticle) × 100 %.

1.2 Growth factors release study and pharmacokinetic studies *in vivo*

Total protein and growth factors release from RMBN were determined as previously described.[1] In brief, freeze-dried RMBN was dissolved in DCM. After that, PBS was added to the solution. The sample was subjected to vortex for 5-10 min to isolate proteins from the oil phase to the water phase. After centrifugation, the protein concentration in the water phase was measured by a bicinchoninic acid assay. For growth factor release studies, NPs were incubated in PBS at 37 °C. The supernatant was collected at different time points (24, 48, 72, 96, 120, and 144 h), and the concentrations of various growth factors were determined by enzyme-linked immunosorbent assays (ELISAs). Ten male C57BL/6 mice were randomized into two groups (n = 5 per group) and were intravenously injected with RMBN nanoparticles (both labeled with Cy5.5 fluorophore during NP preparation). At 0.5, 1, 2, 4, 12, 24,

and 48 h, 20 μL whole blood was collected. NP concentrations were determined by UV–vis spectra using a Nanodrop 2000 (Thermo Scientific, USA). Known concentrations of Cy5.5-labeled NPs were mixed with blood to generate a standard calibration curve. The concentrations of NPs in different blood samples were calculated based on the standard curve.

1.3 *Ex vivo* fluorescent imaging for biodistribution of RMBN

Cohorts of normal mice and liver failure mice were sacrificed at 0, 3, and 6 h after nanoparticle injections; major organs were collected for biodistribution studies using *ex vivo* fluorescent imaging (IVIS, Caliper Lifesciences, Waltham, MA).

1.4 Mouse model of ALF induced by APAP and NMBN therapy

Male C57BL/6J mice were maintained under standard conditions (22 °C ± 2 °C, 50% ± 10% relative humidity, 12 h light/dark diurnal cycles) with ad libitum access to water and a standard rodent chow diet.[4] After one week of adaptation, mice were divided into four groups treated as follows: CON mice: saline; APAP model mice: APAP+PBS; APAP+NAC: APAP+NAC; NMBN: APAP+NMBN. APAP was dissolved in warm saline solution (55–60 °C) and cooled to 37 °C before administration. APAP-induced ALF mice models were constructed, PBS, NAC, or NMBN nanozymes were immediately injected into the mice through the tail vein for 1 h, followed by APAP injection. Mice were euthanized 24 h post APAP injection, and blood samples and the entire liver were collected immediately for further measurements.

For biodistribution analysis, mice were euthanized after 0, 3, 6 h post-intravenous injections of NMBN.

1.5 Mortality and Biochemical analysis

The survival rate of animals was monitored during the 40 h after GalN/LPS treatment. The enzyme activities of plasma alanine aminotransferase (ALT), aspartate aminotransferase (AST), alkaline phosphatase (ALP), total bilirubin (TBIL) and lactate dehydrogenase (LDH) were measured by corresponding commercial assay kits (Nanjing Jianchen Bioengineering Institute, Nanjing, China)

1.6 Histology analysis

Histopathological changes of liver were observed using haematoxylin and eosin (H&E)

staining as described previously.[5] Briefly, the liver tissues from same lobe were fixed in 4% phosphate-buffered paraformaldehyde for 24 h, then embedded in paraffin wax and cut into 4 μm-thick sections, and stained with H&E. Histological changes were evaluated and captured by an Olympus CX31 light microscope (Olympus Corp., Japan).

1.7 Measurement of oxidative stress parameters and cytokines

Liver tissue of each group was weighted and homogenized in cold radioimmunoprecipitation assay buffer (RIPA buffer, Beyotime Institute of Biotechnology, Nanjing, China) to prepare 10% (wt/v) liver homogenate. After centrifugation at 12,000 g for 20 min at 4°C, the resulting supernatant was collected to measure hepatic oxidative stress parameters, including the hepatic levels of thiobarbituric acid reactive substances (TBARS), superoxide dismutase (SOD), activities of catalase (CAT), total antioxidant capacity (T-AOC), and reduced glutathione (GSH) were measured by using commercially available kits (Nanjing Jiancheng Bioengineering Institute, Nanjing, China) according to the manufacturer's instructions. To measure hepatic cytokines, the supernatant mentioned above was also subjected to determine the hepatic levels of TNF-α, IL-6, IL-β, IL-17A, and MCP-1 by their Mouse Enzyme-Linked Immunosorbent Assay (ELISA) MAX™ Standard kits (BioLegend Inc., San Diego, CA, USA), respectively, according to the manufacturer's instructions. The total protein in liver was quantified using a Pierce™ BCA Protein Assay Kit (Thermo Fisher Scientific Inc., Rockford, IL, USA). All values were normalized to hepatic total protein.

1.8 Terminal dUTP nick-end labeling (TUNEL) staining

For the detection of apoptosis-positive cells in liver sections, a terminal deoxynucleotidyl transferase-mediated deoxyuridine 5-triphosphate (dUTP) nick end labeling (TUNEL) assay was performed using ApopTag ®Plus In Situ Apoptosis Fluorescein Detection Kit (S7111, EMD Millipore Corporation, Billerica, MA, USA) according to manufactures' protocol. Hepatic cryostat sections (8 μm) were fixed with 4% paraformaldehyde for 15 min, and incubated with green fluorescein labeled dUTP solution for 1 h at 37 °C. The apoptotic cells were photographed in randomly selected fields from each slide at a magnification of × 400.

1.9 Caspase-3, Caspase-8 and Caspase-9 activity

Caspases activities were measured with a colorimetric commercial kit (Beyotime Institute of Biotechnology, Nanjing, China). Livers were homogenized in lysis buffer with a dounce homogenizer. Reactions were carried out on a standard amount of protein at 37 °C for 2 h, and the optical density was measured on a microplate reader at a wavelength of 405 nm.

1.10 Immunofluorescence assay

Immunofluorescence analysis was performed as described previously.[5] Briefly, cryostat sections of liver samples were fixed with cold acetone for 10 min. After permeabilization and blocking the endogenous peroxidase, the sections were incubated with anti-mouse F4/80 antibody (1:100, AbD Serotec, Raleigh, NC, USA) and anti-mouse CD206 antibody (1:200, Bioworld Techology, Saint Paul, MN, USA) overnight at 4 °C, followed by incubation with a horseradish peroxidase-conjugated anti-rabbit IgG (Life Technologies, Carlsbad, CA, USA) for 1 h at room temperature. Nuclei were counterstained with 4',6-diamine-2-phenylindole dihydrochloride (DAPI). The fluorescence was examined and photographed with a disk scanning unit confocal imaging system (Olympus, Tokyo, Japan).

1.11 Immunoblot assay

Protein concentrations were determined using a Pierce™ BCA Protein Assay Kit (Thermo Fisher Scientific Inc., Rockford, IL, USA). Equivalent amount (40 μg) of liver protein samples in loading buffer was subjected to 8-10% sodium dodecyl sulfate-polyacrylamide gel electrophoresis. Proteins in the gels were transferred into polyvinylidene difluoride membrane (Millipore Corp., Bedford, Massachusetts, USA), and blocked with 5% non-fat dry milk, the membranes were incubated with specific primary antibodies including caspase-3, cleaved caspase-3, caspase-9, cleaved caspase-9, Bax, Bcl2, c-jun, p-c-jun, JNK, p-JNK, ERK, p-ERK, Arg-1, cyclin A2, cyclin D1, PCNA, and GAPDH (Cell Signaling Technology, Inc., Beverly, MA, USA) independently overnight at 4 °C, and then incubated with corresponding horseradish-peroxidase-conjugated secondary antibodies.

Supplementary Figures

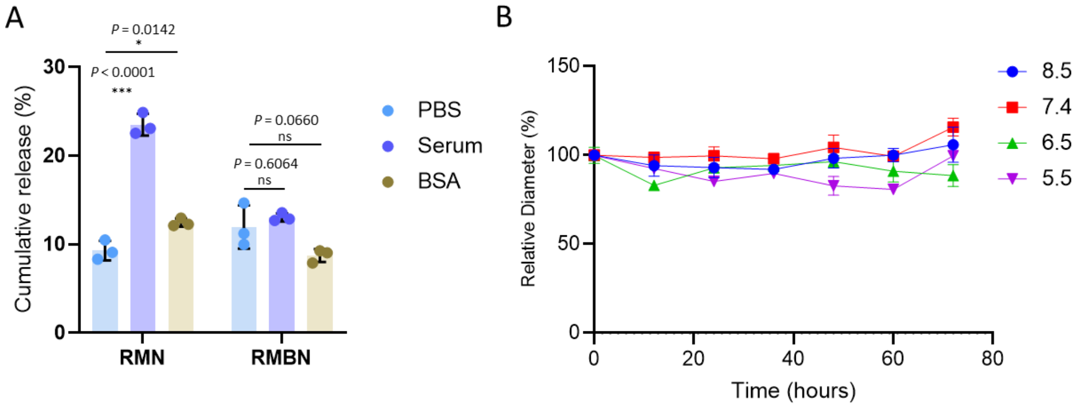

Figure S1. Stability of the RMBN in serum, alkaline and acidic environments. (A) Cumulative release of Rhein from RMBN and RMN when exposed to serum for 1 hour. $*P < 0.05$, $**P < 0.01$, $***P < 0.005$, $^{ns}P > 0.05$, compared with the PBS group. **(B)** Size change of RMBN in different pH solutions (pH 8.5, 7.4, 6.5, and 5.5) at 4°C temperature (n = 3).

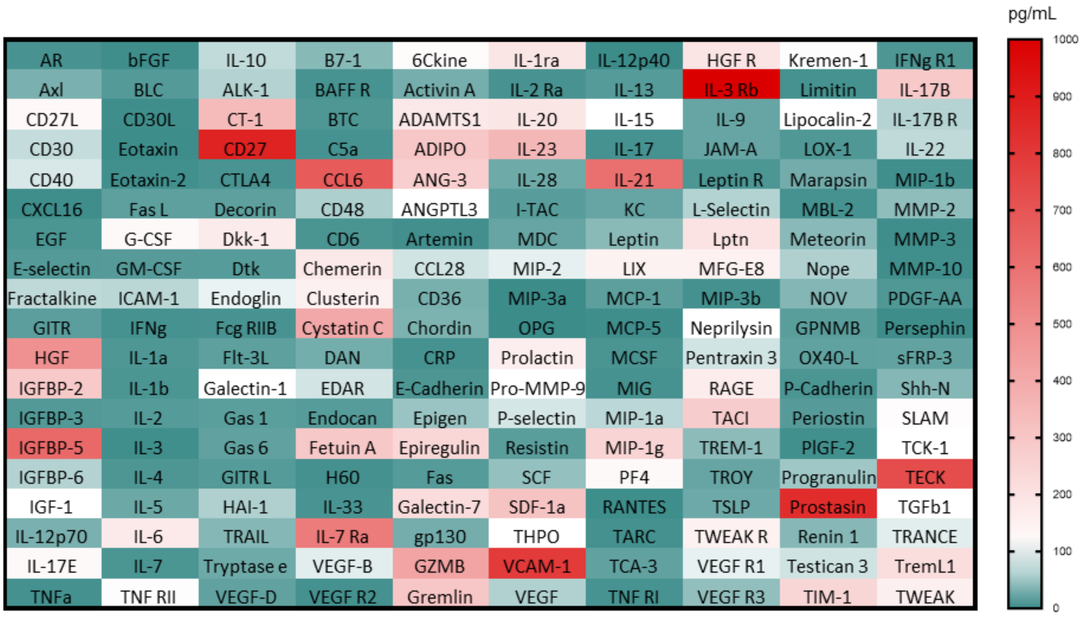

Figure S2. Characterization of representative MSC-secreted factors by protein array. Characterization of MSC-secreted factors was assessed using the RayBio Quantibody® Mouse Cytokine Array 4000 Kit (RayBiotech, USA). Relative concentration of each factor was evaluated using the dedicated software from RayBiotech on the membrane containing internal controls for reference.

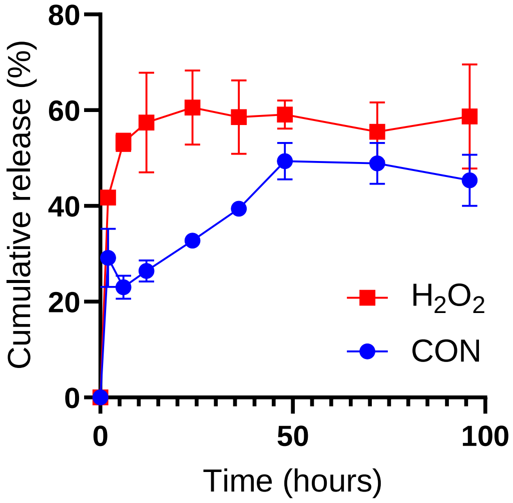

Figure S3. H_2O_2 responsive drug release of RMBN. Cumulative release of Rhein from RMBN when exposed to 10 mM H_2O_2 and PBS (CON).

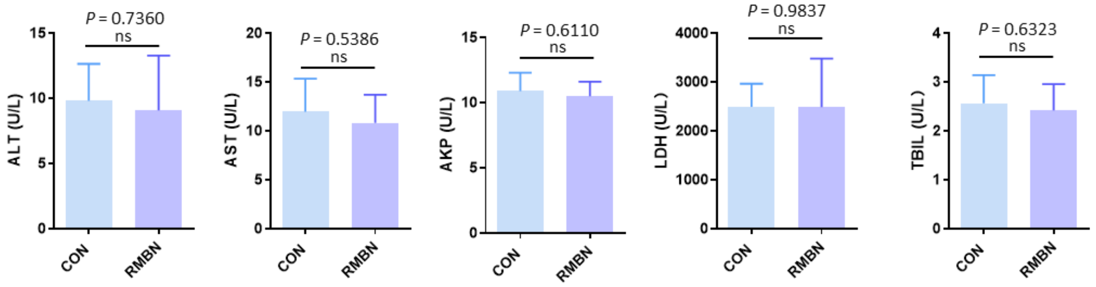

Figure S4. Effects of RMBN on hepatology parameters, including serum ALT, AST, AKP, LDH, and TBIL. *P* values were calculated using T-test.

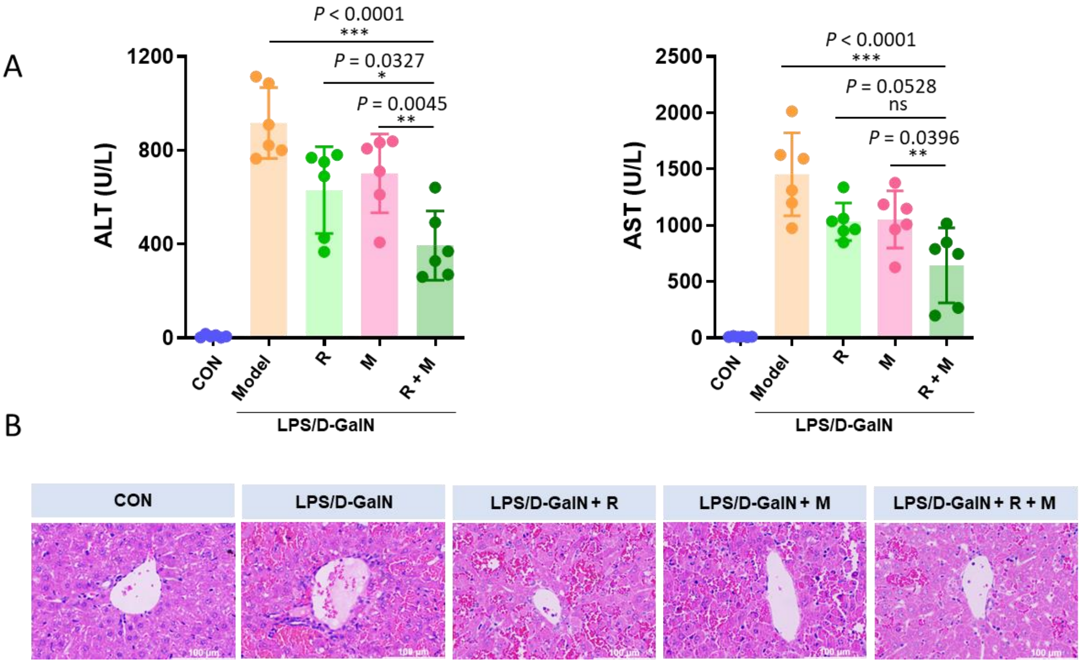

Figure S5. Synergistic effect of the combination of Rhein and MSC-conditioned media in LPS/GalN-induced murine models of ALF. (A) Effects of Rhein and MCM on hepatology parameters serum ALT and AST. (B) H&E staining of representative

liver of mice after the post-injection of Rehin and MCM. R = Rhein; M = MCM, scale bar = 100 μm. Data are expressed as means ± SD (n = 6), *P* values were calculated using a one-way ANOVA with Tukey's multiple comparison test.

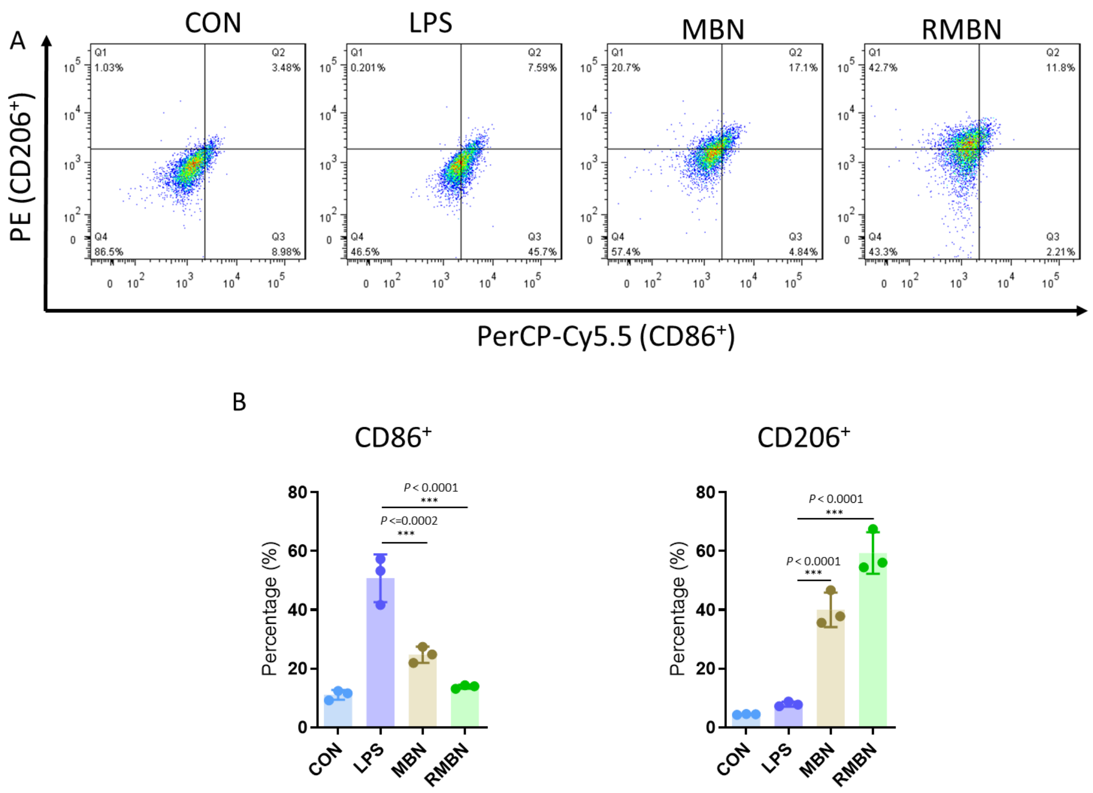

Figure S6. Effect of RMBN on M1 and M2 macrophage differentiation. (A) Flow cytometry analysis of $CD206^+$ and $CD86^+$ expression of RAW 264.7 cells treated with LPS (control), MBN, and RMBN. (B) Statistical histogram of M1 ($CD86^+$) or M2 ($CD206^+$) type macrophages after treatment.

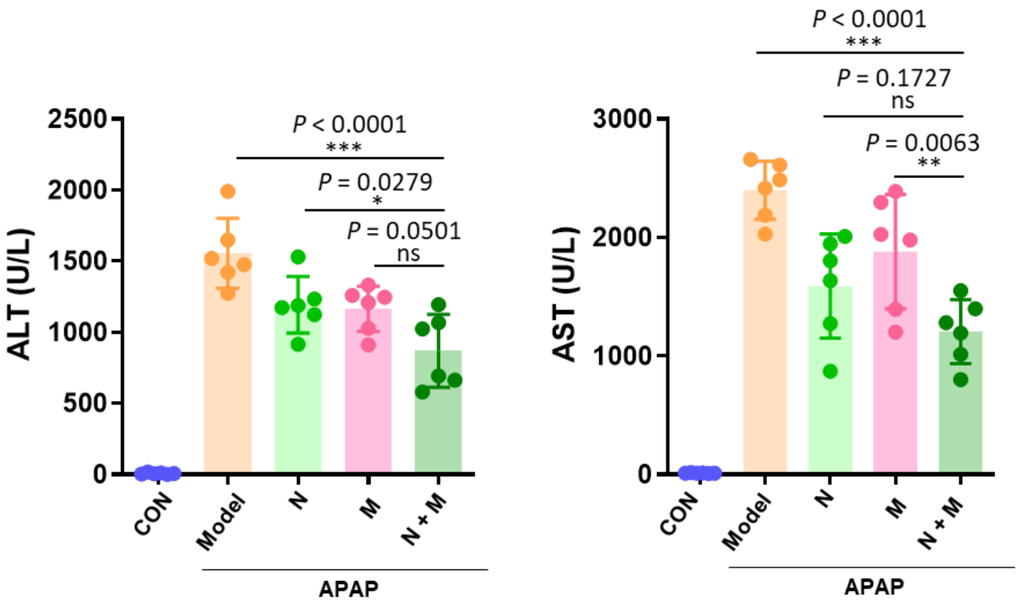

Figure S7. Synergistic effect of the combination of NAC and MCM in APAP-induced mimic ALF. Data are expressed as means ± SD (n = 6), *P* values were calculated using a one-way ANOVA with Tukey's multiple comparison test.

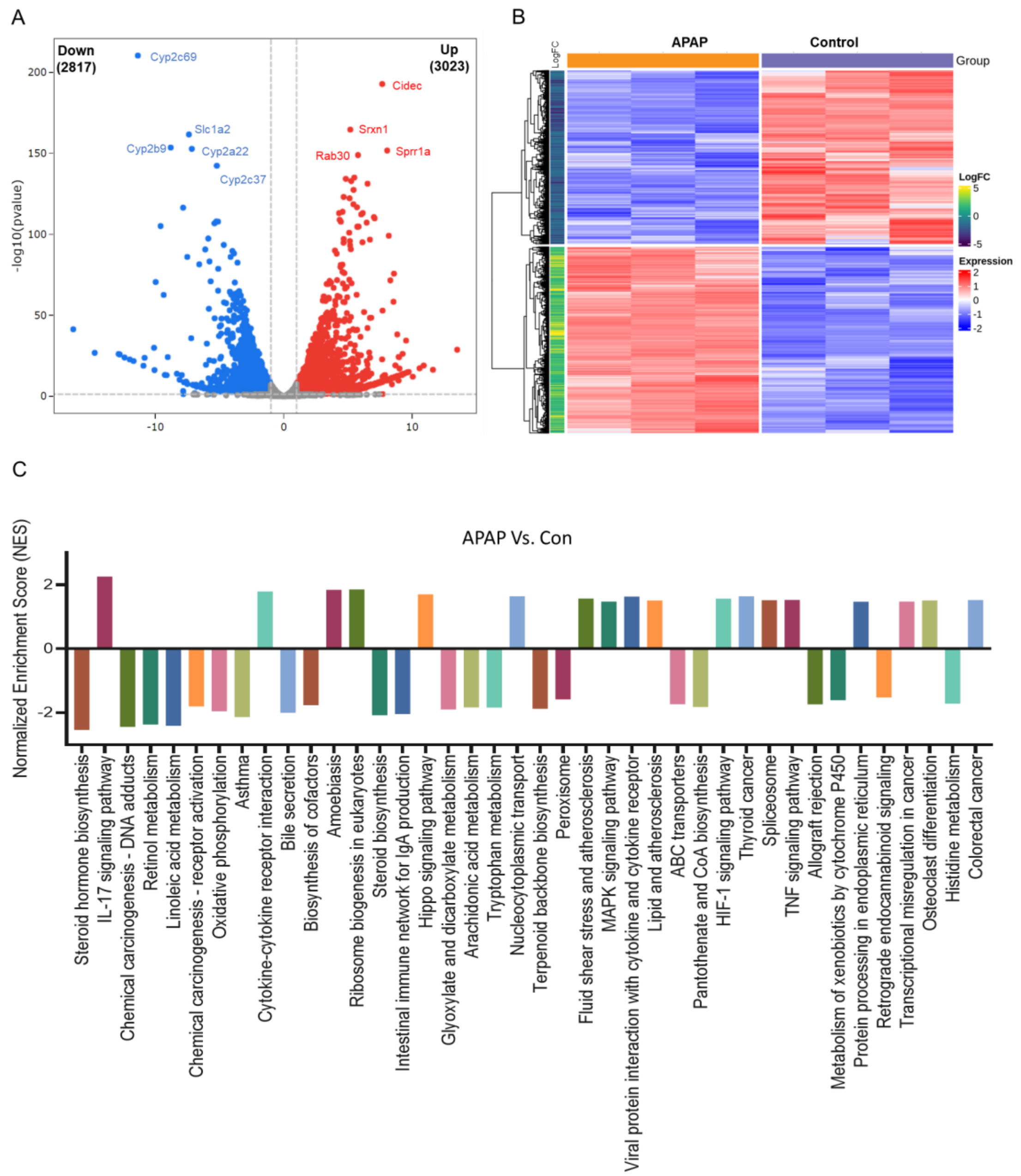

Figure S8. Characterization of APAP-induced mimic ALF. (**A**) Cluster analysis of the differential expressed genes. NMBN (APAP+NMBN) vs. vehicle (APAP) group. (**B**) Volcano plot of the differential expressed genes. NMBN vs. vehicle group. (**C**) Enrichment analyses of the differential expressed genes using Gene Ontology (GO) analysis. APAP vs. Control group.

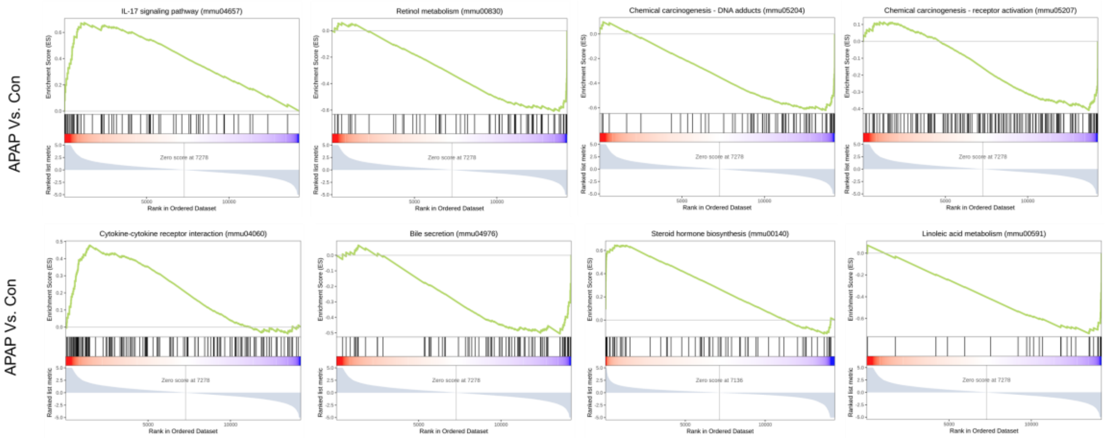

Figure S9. Gene Set Enrichment Analysis (GSEA) of the differential expressed genes of representative pathways in the liver of mimic ALF mice. APAP vs. Control group.

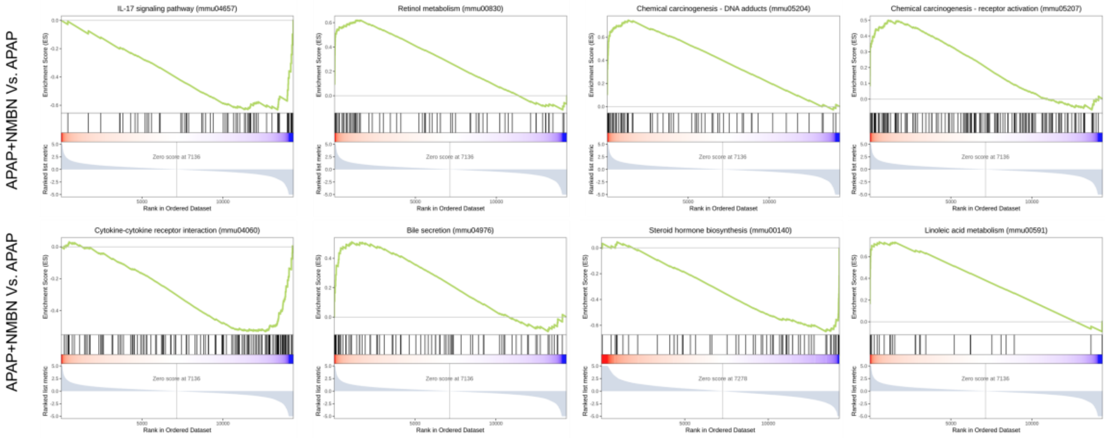

Figure S10. Gene Set Enrichment Analysis (GSEA) of the differential expressed genes of representative pathways in the liver of mimic ALF mice. NMBN+APAP vs. APAP group.

References

(1) Liang, H.; Huang, K.; Su, T.; Li, Z.; Hu, S.; Dinh, P.-U.; Wrona, E. A.; Shao, C.; Qiao, L.; Vandergriff, A. C. Mesenchymal stem cell/red blood cell-inspired nanoparticle therapy in mice with carbon tetrachloride-induced acute liver failure. *Acs Nano* **2018**, *12* (7), 6536-6544.

(2) Liu, M.; Dasgupta, A.; Koczera, P.; Schipper, S.; Rommel, D.; Shi, Y.; Kiessling, F.; Lammers, T. Drug loading in poly (butyl cyanoacrylate)-based polymeric microbubbles. *Molecular pharmaceutics*

2020, *17* (8), 2840-2848.

(3) Shoma Suresh, K.; Bhat, S.; Guru, B. R.; Muttigi, M. S.; Seetharam, R. N. A nanocomposite hydrogel delivery system for mesenchymal stromal cell secretome. *Stem cell research & therapy* **2020**, *11* (1), 1-14.

(4) Feng, Q.; Xu, H.; Pan, X.; Geng, S.; Qian, H.; Wang, C.; Li, Y.; Qin, J.; Wu, Y.; Zhou, B. Antioxidation and Anti-Inflammatory Activity of Prussian Blue Nanozymes to Alleviate Acetaminophen-Induced Acute Liver Injury. *ACS Applied Nano Materials* **2023**.

(5) Feng, R.; Ma, L.-J.; Wang, M.; Liu, C.; Yang, R.; Su, H.; Yang, Y.; Wan, J.-B. Oxidation of fish oil exacerbates alcoholic liver disease by enhancing intestinal dysbiosis in mice. *Communications Biology* **2020**, *3* (1), 481.
